# Tuning the Through-Plane Lattice Thermal Conductivity in van der Waals Structures through Rotational (Dis)ordering

**DOI:** 10.1021/acsnano.3c09717

**Published:** 2023-12-08

**Authors:** Fredrik Eriksson, Erik Fransson, Christopher Linderälv, Zheyong Fan, Paul Erhart

**Affiliations:** † Department of Physics, Chalmers University of Technology, SE-41296 Gothenburg, Sweden; ‡ College of Physical Science and Technology, Bohai University, Jinzhou 121013, People’s Republic of China

**Keywords:** Thermal conductivity, van der Waals materials, Molecular dynamics, Moiré structures, Machine-learning potentials, Atomic-scale modeling

## Abstract

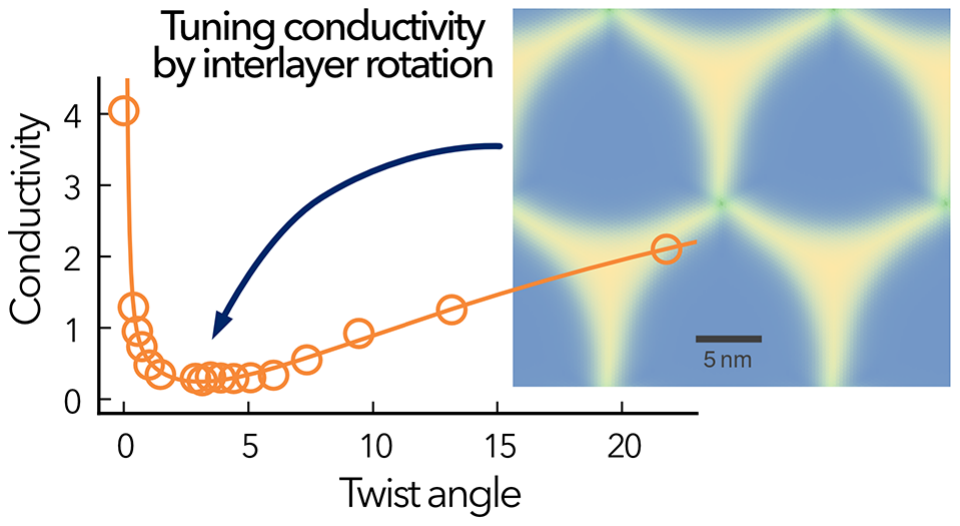

It has recently been demonstrated that MoS_2_ with irregular interlayer rotations can achieve an extreme anisotropy in the lattice thermal conductivity (LTC), which is, for example, of interest for applications in waste heat management in integrated circuits. Here, we show by atomic-scale simulations based on machine-learned potentials that this principle extends to other two-dimensional materials, including C and BN. In all three materials, introducing *rotational disorder* drives the through-plane LTC to the glass limit, while the in-plane LTC remains almost unchanged compared to those of the ideal bulk materials. We demonstrate that the ultralow through-plane LTC is connected to the collapse of their transverse acoustic modes in the through-plane direction. Furthermore, we find that the twist angle in periodic moiré structures representing *rotational order* provides an efficient means for tuning the through-plane LTC that operates for all chemistries considered here. The minimal through-plane LTC is obtained for angles between 1 and 4° depending on the material, with the biggest effect in MoS_2_. The angular dependence is correlated with the degree of stacking disorder in the materials, which in turn is connected to the slip surface. This provides a simple descriptor for predicting the optimal conditions at which the LTC is expected to become minimal.

## Introduction

Understanding the atomic-scale dynamics of materials is important from both conceptual and practical vantage points. They are not only fundamental to the thermodynamic and kinetic properties of materials but also strongly affect electronic transport and optical response. The lattice thermal conductivity (LTC) in particular is important for applications in, e.g., thermoelectrics and thermal management.^1^ In the latter case, anisotropic thermal conductors have been proposed as an efficient means for removing waste heat.^2−4^

**Figure 1:**
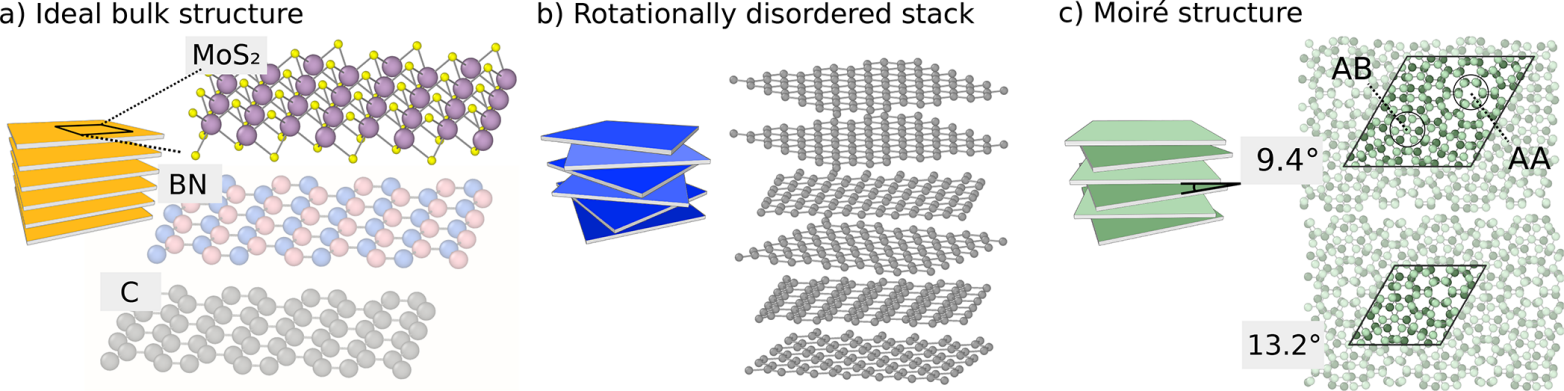
Van der Waals structures consist of monolayers with strong intralayer and weak (vdW-mediated) interlayer interactions. (a) Ideal bulk structures of MoS_2_, BN, and C (graphite) are characterized by perfect registry between the layers (only monolayers shown). (b) In rotationally disordered stacks the twist angles between the monolayers are random. (c) In (bulk) moiré structures every other layer is rotated with the same twist angle. They can serve as simple model systems, providing insight into the mechanisms giving rise to ultralow through-plane LTC and large anisotropy.

Van der Waals (vdW) materials consist of quasi-two-dimensional layers with strong intralayer and weak (vdW-mediated) interlayer interactions. In the ideal bulk form of, e.g., MoS_2_, C (graphite), or BN, the layers are highly ordered, typically with a two-layer repetition period (Figure 1a). In disordered vdW materials, on the other hand, the orientation (i.e., the rotational angle) between the layers is random (Figure 1b). Such materials have naturally high anisotropy ratios, a property that is of potential interest, especially for thermal management applications.^5−9^ Yet the artificial synthesis of materials with comparable anisotropies and through-plane conductivities of less than 0.1 W m^–1^ K^–1^ was accomplished only recently.^10^ This progress has been enabled by synthesis routes that allow manipulation of the angles between individual layers in many-layer samples.^11^

It is well-known that interlayer rotations in two-dimensional vdW-bonded structures lead to the emergence of moiré patterns (Figure 1c) and unusual properties.^12−18^ The twist angle provides an additional (structural) degree of freedom that can be used, for example, to induce superconductivity in bilayer graphene.^19,20^ Given the effect of the twist angle on electronic properties, it is natural to ask whether it can also be used to manipulate the LTC of these materials. If the goal is to maximize the LTC anisotropy, lowering the through-plane LTC is key, as the in-plane LTC is bounded from above by the LTC of the corresponding monolayer.

Several mechanisms may play a role in lowering the through-plane LTC of vdW structures in general,^8,21^ including interlayer rotations.^22−25^ Interlayer rotations cause the atoms in adjacent layers to be pushed out of registry. This drastically reduces the shear resistance and is manifested in the localization of the corresponding transverse acoustic (TA) phonon modes.^10,25−27^ Moreover, with decreasing twist angle the moiré cell grows, leading to more extended displacement patterns. At the same time, there is a limit to the disorder associated with these displacements, since for sufficiently small angles the layers reconstruct into regions of the energetically favored bulk stacking that are separated by domain walls.^18,28−31^ This reconstruction is governed by the intrinsic properties of the material such as the elastic constants and the interlayer potential energy landscape. The interplay of these factors can be expected to lead to a minimum in the through-plane LTC as a function of the twist angle. Quantitative assessments of these effects require, however, accurate and predictive atomic-scale simulations that can guide future experimental studies.

For materials with relatively high symmetry and modest unit cell sizes, the LTC can be accurately predicted and analyzed in the framework of the Peierls–Boltzmann transport equation (PBTE) using force constants calculated via electronic structure methods such as density functional theory (DFT). Due to the scaling of both the PBTE and the electronic structure calculations, this approach becomes, however, prohibitive for materials with larger unit cells and/or lower symmetry. This challenge can be overcome using Green–Kubo (GK) methods in conjunction with molecular dynamics (MD) simulations, which, however, require suitable interatomic potentials.

**Figure 2:**
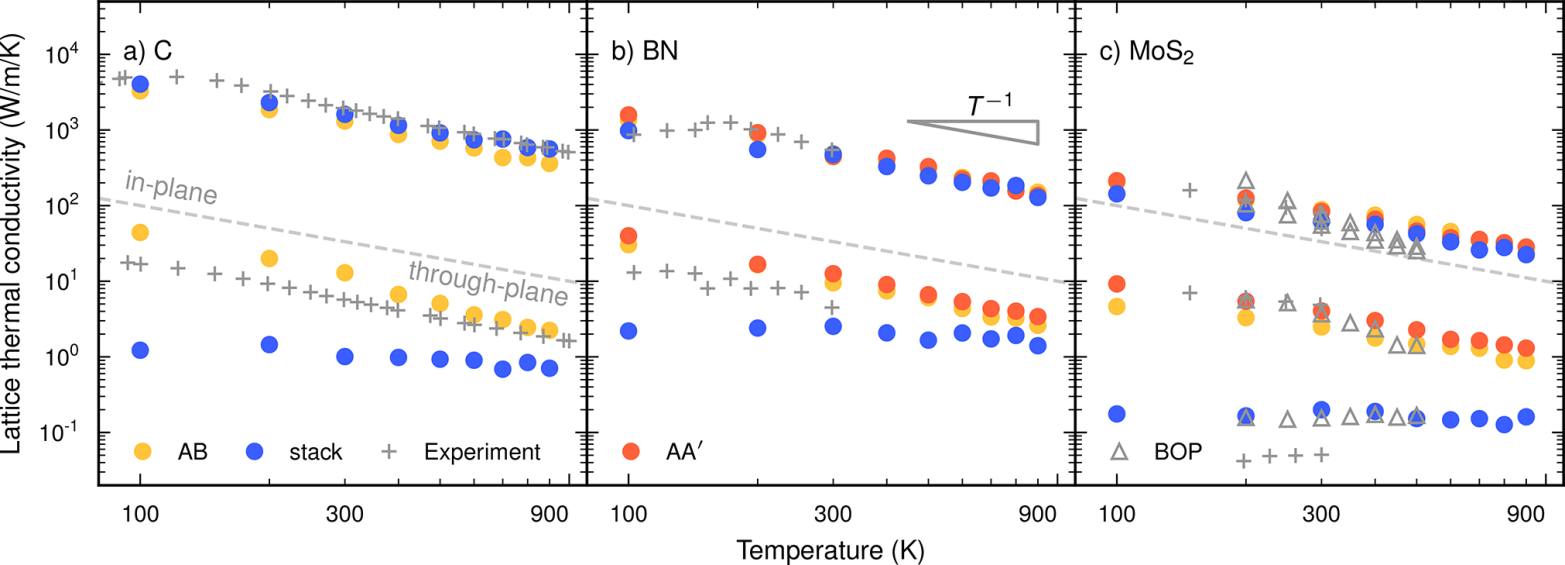
LTC for (a) C, (b) BN, and (c) MoS_2_ as a function of temperature for the ideal bulk systems as well as a rotationally disordered stack system with random interlayer rotations. The dashed gray line separates the in-plane and out-of-plane components of the LTC tensor. The gray plus signs indicate experimental data from ref (32) (C; graphite), ref (33) (BN), and ref (10) (MoS_2_). The triangles in (c) represent results from simulations based on a BOP model from ref (10). The statistical errors for the thermal conductivity are about the size of the markers across all data points.

Here, we employ the GK approach in combination with machine-learning potentials (MLPs) to analyze the LTC for three prototypical vdW materials with interlayer rotations: graphite/graphene (C), hexagonal boron nitride (BN), and molybdenum disulfide (MoS_2_). We focus on two types of three-dimensionally periodic structures that are compared to the ideal bulk structures (Figure 1a): (1) *stacks* with arbitrary rotation angles and small in-plane strains comprising up to 10 layers per unit cell, representing *rotational disorder* (Figure 1b), and (2) *moiré structures* with a single rotation angle, i.e., the primitive cell contains two monolayers with a specific rotation angle, representing *rotational order* (Figure 1c). We show that for all three materials rotational disorder gives rise to a systematic and substantial reduction in the through-plane LTC without strongly affecting the in-plane conductivity. In all cases, we find that the stacks display glass-like conduction with the largest LTC anisotropy appearing in C, for which we obtain a ratio of over 1000 at room temperature.

Further insight is provided by the dependence of the LTC on the twist angle in periodic moiré structures, which we relate to the atomic level reconstructions. The latter connection enables a particularly simple interpretation of the angular dependence of the LTC in terms of the slip surfaces of the different materials. Our results demonstrate that rotational disorder can be used for manipulating the LTC in layered materials that is largely agnostic to chemistry and can provide insight into the underlying mechanisms. We expect that these insights can be exploited, e.g., for developing materials for heat management in integrated circuits, and more generally contribute to not only understanding but also controlling thermal conduction at the nanoscale.

## Results and Discussion

### LTC in Bulk and Disordered Stacks

#### Carbon

To begin, we consider the temperature dependence of the LTC for the stack structure and compare it with the ideal bulk structures for the case of carbon (Figure 2). For the in-plane LTC of the ideal bulk structure (AB, graphite) the simulations are in very good agreement with experimental data.^32^ This applies not only for the MLP based on van der Waals density functional with consistent exchange (vdW-DF-cx) shown here but also for models based on the PBE+D3 and strongly constrained and appropriately normed (SCAN) exchange-correlation (XC) functionals, as shown by PBTE calculations (Figure S9). For the through-plane LTC the simulations somewhat overestimate the experimental data for temperatures below approximately 600 K. This is expected, as the through-plane LTC is not only more difficult to measure but also much more sensitive to sample purity and (small) variations in the aspect ratio. This is also evident from the comparison with the PBTE results for the models based on other XC functionals, which overestimate the aspect ratio and underestimate the through-plane LTC (Figure S9).

The vdW-DF-cx method and accordingly the vdW-DF-cx-based MLP achieve good overall agreement with the structural parameters as well as experimental data, demonstrating that they capture the vibrational excitations that govern thermal conduction in this material.

Moving on to the stack system with rotational disorder, one observes a substantial drop in the through-plane LTC while the in-plane LTC remains at the level of the ideal bulk system. For example, at 300 K the through-plane LTC is reduced by more than a factor of 10, leading to an anisotropy ratio between the fast and the slow LTC components of more than 1000. The LTC is moreover constant over the temperature range considered here, a behavior commonly observed in glasses.^78^ As further discussed below, this can be understood as the phonon mean free path for through-plane transport being approximately limited to the interlayer distance.

It is noteworthy that the in-plane LTC for the stack even exceeds that of graphite. This effect can be attributed to the weaker coupling between layers, which affects the flexural modes and thereby the LTC. This effect is also apparent in the larger in-plane LTC of graphene sheets compared to graphite.^34−37^

#### Boron Nitride

In BN the behavior of the thermal conductivities is qualitatively the same as that for C (Figure 2b). While there are two types of ideal bulk stackings, AA′ and AB, the difference in LTC between these two structures is minimal. The agreement with experimental data^33^ for the in-plane conductivity is very good, and the LTC falls off with *T*^–1^. For the through-plane conductivity, the simulations yield slightly higher conductivities than experiment, equivalent to and for similar reasons as in the case of C. Also the behavior of the LTC for the stack system is similar, showing the same kind of temperature-independent conductivity. The reduction in the through-plane LTC when going from the ideal to the stack system is, however, notably smaller than that in the case of C, leading only an anisotropy ratio of about 200 at 300 K.

#### Molybdenum Disulfide

For the ideal bulk structure of MoS_2_ both the in-plane and through-plane conductivities are in very good agreement with experimental data (Figure 2c)^10^ and, as in the case of BN, the LTCs are practically the same for AA′ and AB structures. As for the other two materials, the in-plane LTC for the stack system is almost unchanged compared to that of the ideal bulk system, while the through-plane LTC exhibits a glass-like temperature dependence, achieving an anisotropy ratio of about 300 at 300 K.

The calculated through-plane LTC for the stack is notably higher compared to experiments.^10^ This is likely due to other effects, besides the stacking, being at play in the experimental study that are not captured in the simulations, including, e.g., the presence of defects^38,39^ and the contribution of interface resistivity in the experimental devices.

Here, we also include a comparison with LTC data obtained previously^10^ via MD simulations using a bond-order potential (BOP) model.^40,41^ While the latter yields a somewhat steeper temperature dependence for the in-plane conductivity, the results are overall very close, including, in particular, the through-plane LTC for the stack system. This agreement is noteworthy, given that the neuroevolution potential (NEP) models used in the present work and the BOP model employ very different functional forms and were constructed by using different reference data and design principles. This goes to show that the effect revealed here is not sensitive to the specifics of the underlying model but rather an intrinsic feature of the material and structure.

### Rotational Disorder in the Phonon Dispersion

**Figure 3:**
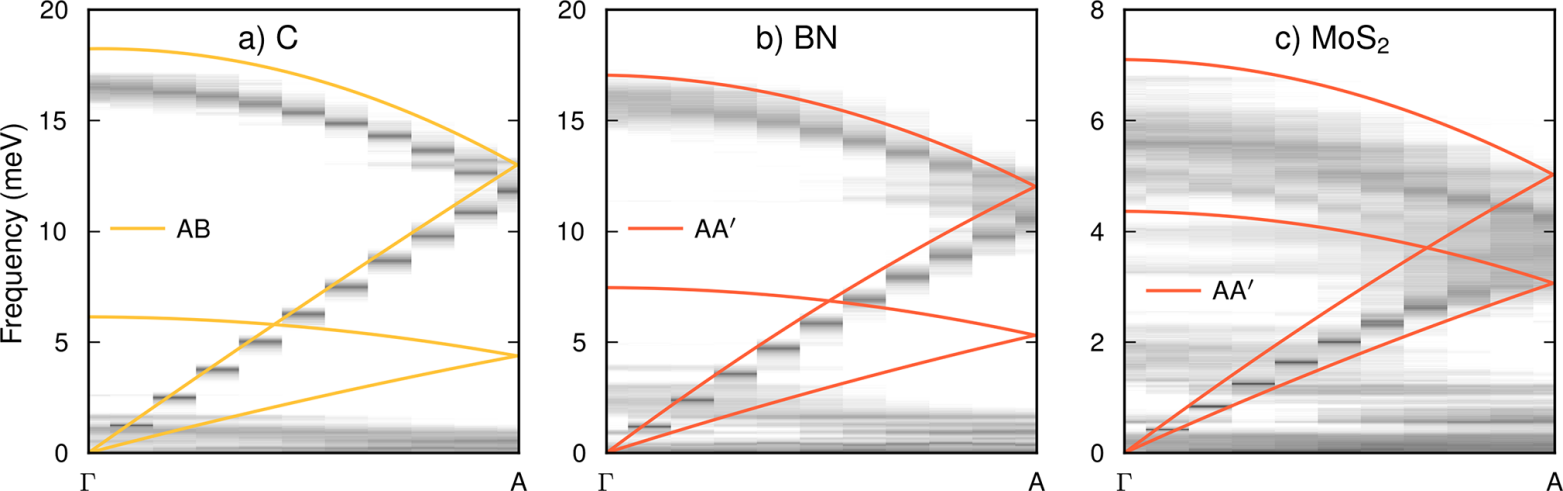
Phonon dispersions obtained via mode projections from MD simulations for ideal bulk (lines) and stack structures (heat map) at 300 K for (a) carbon, (b) boron nitride, and (c) molybdenum disulfide along the Γ → A direction ([0,0,1]). The heat maps show the natural logarithm of the velocity power spectra obtained by projection onto the normal modes of the respective ideal bulk structure (see Methods for details).

In order for a mode to contribute to conduction in the through-plane direction, it must have a nonzero group velocity component in the *z* direction, which applies for modes that fall within a rather narrow cone along the Γ–A path.^42^ To reveal the microscopic mechanisms that lead to the dramatic reduction in the through-plane LTC in the stack structures, it is therefore instructive to inspect the vibrational spectra along Γ–A. This analysis (see Mode Projection in Methods) reveals that the dispersion of the longitudinal acoustic (LA) modes is only very weakly affected when rotational disorder is introduced (Figure 3). At the same time, one observes a collapse of the TA and the lowermost transverse optic (TO) modes in all stack systems. In other words, these modes soften significantly and the frequencies become nearly independent of the momentum vector, as previously shown^10^ in the case of MoS_2_ using a BOP model.^40^

One can understand the collapse of the TA modes as being related to a large reduction of the shear resistance. The latter arises because the interlayer rotations push the layers out of registry, which reduces the energy barriers that need to be overcome to shear neighboring layers relative to each other.

A further important observation is that the phonon lifetimes of the LA (and the longitudinal optic (LO)) modes can drop by as much as 2 orders of magnitude when going from the ideal bulk to the stack structures (Figure S8). Interestingly, one can show that reducing the frequencies of the TA modes while leaving the phonon–phonon interaction (i.e., the third-order force constants) unchanged is sufficient to achieve a dramatic drop in the through-plane conductivity *without* introducing explicit disorder (Supplementary Note S1 and Figure S10). This demonstration is distinct from the observation that a rescaling of the (entire) interaction potential leads to a negative correlation between the in-plane and through-plane conductivities.^21^

### LTC Reduction in Terms of Phonon Scattering

To obtain a conceptually intuitive understanding of the low through-plane LTC in rotationally disordered systems, recall that according to the linearized solution of the PBTE^43^ the LTC is given by a summation over all phonon modes
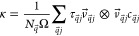
1Here,  is the lifetime,  is the group velocity, and  is the mode-specific heat capacity, which in the classical limit is a constant, while q⃗ and *j* indicate the phonon momentum and branch. Finally,  is the number of q⃗ points included in the summation and Ω is the unit cell volume.

In this picture, a drop in the LTC can thus result from a reduction in the lifetimes or group velocities. As the group velocity of the heat-carrying longitudinal modes is only weakly affected by interlayer rotations (Figure 3), the majority of the reduction must be attributable to a reduction of the lifetimes, which is consistent with our analysis (Figure S8).

Due to the large anisotropy in vdW structures, the phonon modes in these materials can be separated into two distinct regions in the Brillouin zone, predominantly contributing to the in-plane and out-of-plane LTCs, respectively.^42^ In the former region, the group velocities are close to zero in the through-plane direction and the modes are monolayer-like. The modes in the second set are confined to a narrow cone along Γ–A with in-plane group velocities that are close to zero, contributing very little to the in-plane thermal conductivity. As a result of this separation, the collapse of the TA mode in rotationally disordered stacks and the decrease of the LA mode lifetimes have almost no impact on the in-plane LTC.

### LTC in Rotationally Ordered Systems

**Figure 4:**
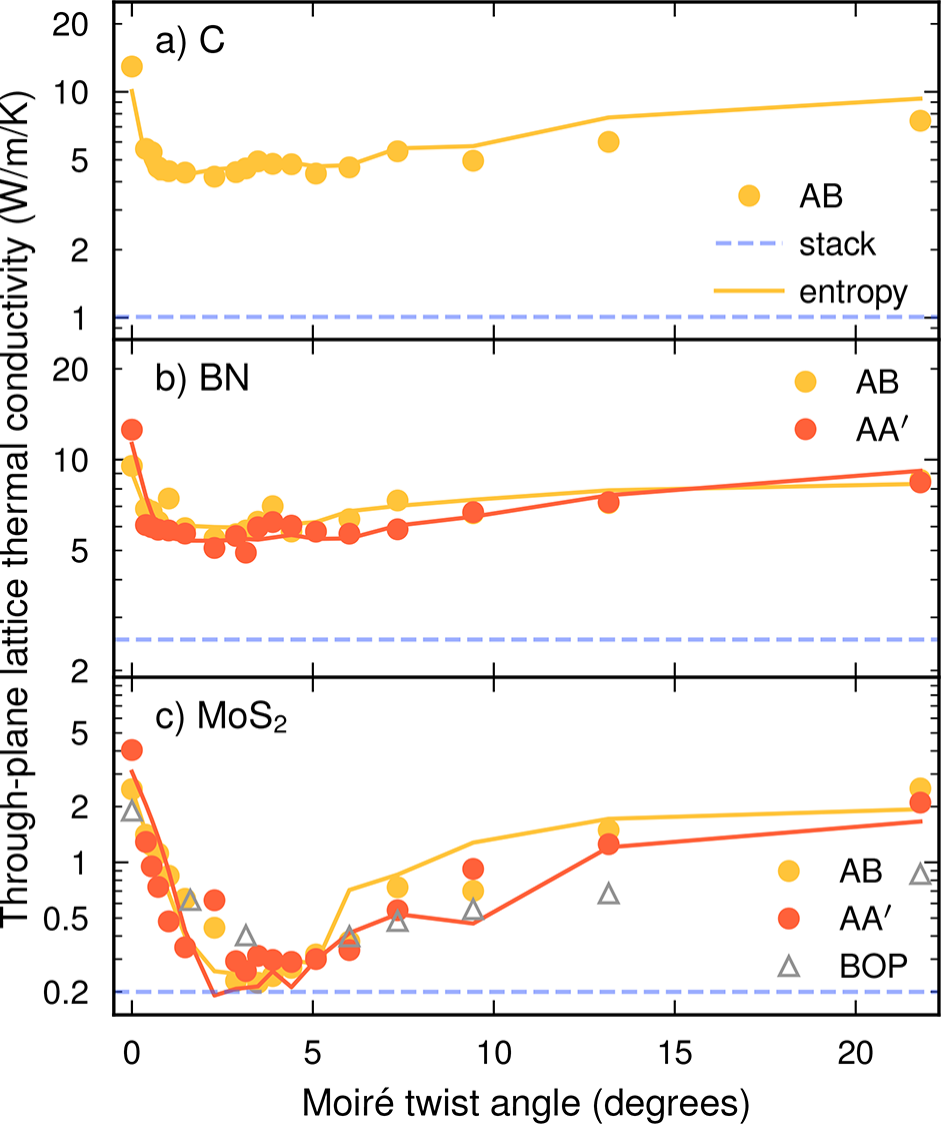
LTCs of moiré structures as a function of twist angle at 300 K in (a) carbon, (b) BN, and (c) MoS_2_. The statistical errors for the thermal conductivity are about twice as large as the markers across all data points. The negative entropy −*S* of the stacking order parameter (see eq 2) is shown as solid lines using an arbitrary *y*-scale, demonstrating the correlation between stacking disorder and low LTC. The dashed lines indicate the LTC of the stack systems (compare Figure 2).

To gain further insight into the reduction of the through-plane LTC in *rotationally disordered* stacks of layers, it is useful to study the dependence of the LTC on the rotation angle in *rotationally ordered* systems. For all three materials and both stackings, we observe that there exists a minimum in the LTC between 0 and 5° (Figure 4). In MoS_2_ the minimum is very pronounced, and for twist angles of around 3° the through-plane LTC approaches the same value as for the stack. For C and BN the minima are wider and less pronounced, and the minimal LTC values are still notably above the values obtained in the respective stacks.

The theoretical lower limit for the LTC in dense materials is reached when the mean free path available of the heat-carrying phonon modes becomes comparable to the interatomic distances.^44^ For example, in the case of turbostratically deposited MoS_2_, which achieves an ultralow LTC in the through-plane direction,^7^ the effective mean free path approaches the interlayer spacing.^26^ While the layer spacing in MoS_2_ is about 6 Å, it only amounts to about 3 Å in C and BN. This suggests that while interlayer rotations with a periodicity of two layers are sufficient to approach the minimal mean free path, longer sequences are required to achieve the same effect in C and BN.

Lastly, it is striking that the through-plane LTC in MoS_2_ obtained with the NEP is in close agreement with the results obtained using a BOP model in terms of both the absolute values and the position of the angle corresponding to the minimal LTC. This provides a further indication that the results obtained here are caused by a generic microscopic mechanism rather than tied to the details of the atomic interaction models.

### Reconstruction in Moiré Structures

The prediction of the LTC from atomic-scale simulations is computationally demanding. It is therefore desirable to identify simpler predictors of the observed behavior. At the first level of abstraction, one can consider the atomic displacement patterns that emerge in the moiré structures.

The interlayer rotations force the atoms in neighboring layers into energetically less favorable stackings (stacking sequences). To minimize the energy, the atoms in each layer then undergo displacements, which gives rise to a reconstruction into regions that are similar to the ideal bulk stackings separated by “domain walls”. The size of each of these regions depends on the material-specific energy landscape (slip surface).

**Figure 5:**
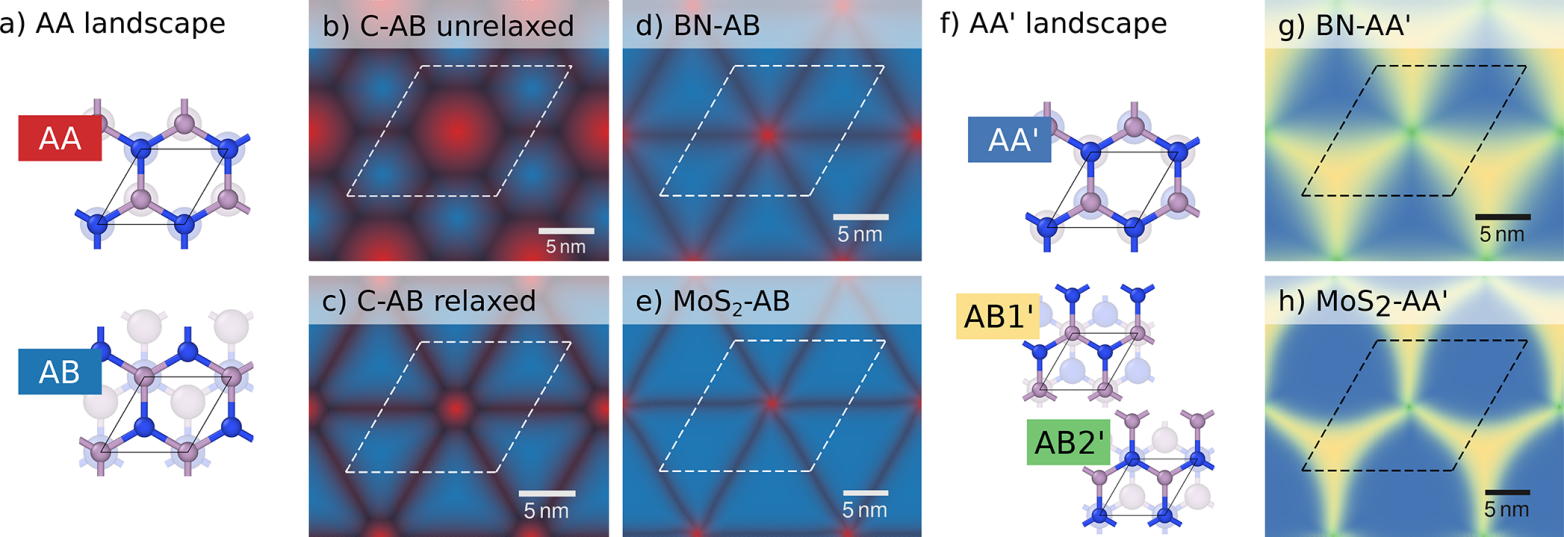
Variation of the local environment with position in a moiré structure with a twist angle of 1.02° for (a–e) AB stacking and (f–h) AA′ stacking. The different colors indicate the similarity with different (ideal) bulk stacking sequences shown in (a, f) obtained via template matching (see Stacking Order Parameter in Methods). A comparison of relaxed (c) and unrelaxed structures (d) shows how reconstruction allows the system to form extended regions of energetically more favorable stacking sequences. The eventual structure is the result of a balance between in-plane strain and domain wall formation. The size of the different regions in the different materials correlates with their respective slip surfaces (Figure 6).

To quantify the size of these different regions, we can define a simple order parameter (see eq 3). For the AB-based moiré structures this reveals extended AB regions separated by domain walls with small AA regions at the domain wall intersections.^45^ The AA′-based structures, on the other hand, feature extended AA′ domains separated by large AB1′ domains and small AB2′ regions at the intersections. The regions with the ideal bulk stacking are most extended for MoS_2_ compared to C and BN (Figure 5).

Using the order parameter α_*i*_ defined in eq 3, we can moreover define a measure for the stacking disorder by estimating the entropy of the probability distribution over the order parameters α as
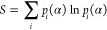
2Here, *p*_*i*_(α) is the probability distribution over α found in a structure. *S* thus measures the relative occurrence of different local stackings in the system.

As shown by the solid lines in Figure 4, the negative entropy −*S* exhibits a very similar angular dependence as the through-plane LTC. This indicates that the LTC is to a large extent correlated with the disorder in the system and conversely that *S* can serve as a simple (and much less expensive) indicator for the angular dependence of the conductivity.

### Slip Surface

It is now natural to ask which material parameters determine the reconstruction in the moiré structures. The latter is driven by the energy gain when forming regions that conform to the low-energy bulk stacking, which needs to be balanced with the cost associated with the geometrically necessary regions with higher energy stacking sequences. Reconstruction requires in-plane and possibly even out-of-plane atomic displacements and thus introduces a local in-plane strain and an associated strain energy. In other words, the reconstruction is induced by interplanar interactions but opposed by intraplanar interactions. The driving force for reconstruction can thus be expected to be larger in materials with a large energy difference between different stacking sequences and small in-plane stiffness (allowing for larger relaxations).

**Figure 6:**
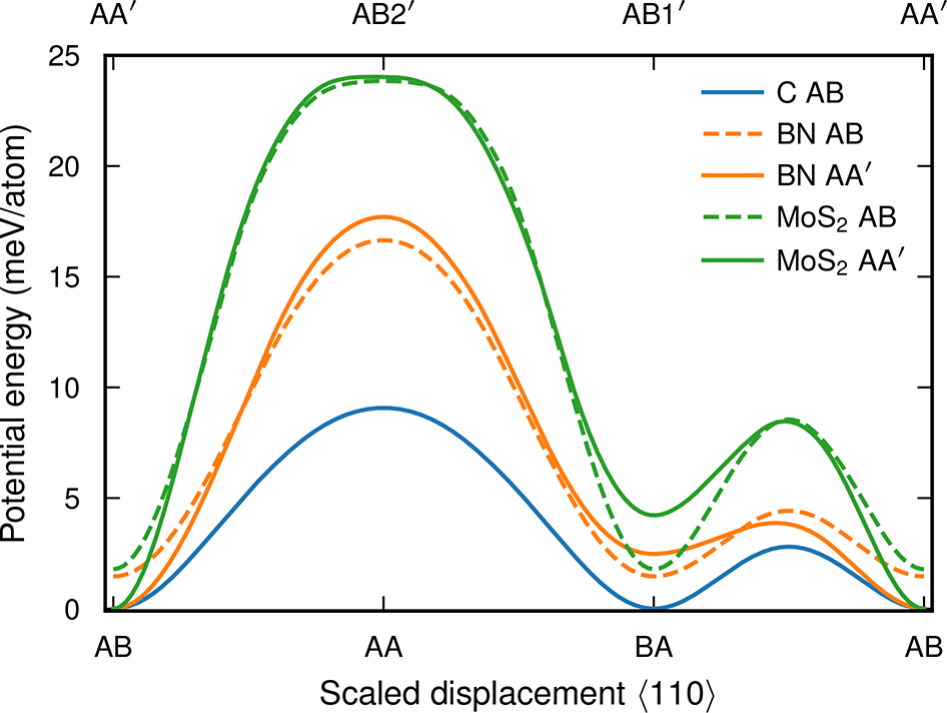
Slip surfaces along the ⟨110⟩ direction. Solid lines indicate the energy surface associated with the respective ground-state structure for each material (AB for C and AA′ for BN and MoS_2_). Dashed lines indicate the slip surfaces based on the AB stacking for BN and MoS_2_. Energies between labeled structures are calculated for geometrically interpolated structures, corresponding to a translation of every other monolayer along the ⟨110⟩ direction. Here, BA refers to a symmetrically equivalent stacking of AB.

The slip surfaces (Figure 6), which provide a picture of the energy landscape for in-plane displacements, show that MoS_2_ exhibits much larger energy differences between the different stackings than C and BN. Furthermore, the in-plane elastic constants (in-plane stiffness) are about 5 times larger in C and BN compared to MoS_2_. Both of these effects contribute to a larger driving force for reconstruction in MoS_2_, and in fact it is for MoS_2_ that one observes the most extended low-energy stacking regions and the most narrow domain walls (Figure 5).

The differences in slip surface, reconstruction, order parameter, and thermal conductivity of the moiré structures in the three materials considered here thus form a coherent picture. Larger energy differences between the different stacking patterns and smaller in-plane stiffness allow for a more extensive reconstruction. The latter leads to a larger entropy in the stacking order parameter, which is in turn correlated with the LTC.

## Conclusions

The results and analysis provided in this study, building on earlier work for MoS_2_,^10^ demonstrate that interlayer rotations in vdW materials can be used for various different chemistries to control the through-plane LTC while leaving the in-plane LTC largely unchanged. For all three materials considered here (C, BN, and MoS_2_) *rotational disorder* in the form of stacks with random interlayer rotations leads to a very substantial reduction in the through-plane LTC (Figure 2), resulting in a very large anisotropy between the through-plane and in-plane conductivities with ratios of about 1000 (C), 200 (BN), and 300 (MoS_2_) at 300 K. In all three cases, the through-plane LTC is practically independent of temperature, indicating a glass-like conduction mechanism with minimal LTC values of about 1 W m^–1^ K^–1^ (C), 2 W m^–1^ K^–1^ (BN), and 0.2 W m^–1^ K^–1^ (MoS_2_). The last value can be compared with experimental data for MoS_2_,^10^ which achieves an even lower level of 57 mW m^–1^ K^–1^. The difference can likely be attributed to the presence of additional defects in the experimental samples and the strong sensitivity of the through-plane LTC to soft vdW-mediated interlayer interactions.

The rotational disorder present in the stack systems causes the collapse of the transverse acoustic modes in the through-plane direction (Figure 3) and a reduction in the lifetimes of both the longitudinal and transverse acoustic modes (Figure S8). This indicates that the mean free paths of the heat-carrying modes become comparable to the interlayer spacing, as expected in the glass limit.

Additional insight is provided by the dependence of the through-plane LTC on the twist angle in *rotationally ordered* moiré structures (Figure 4). For all three materials, one observes a minimum in the through-plane LTC between approximately 1 and 3°, which is most clearly pronounced in MoS_2_. In the latter case the minimal LTC is moreover comparable to the value obtained in the stack system. For C and BN, on the other hand, there is still a notable gap between the minimal LTC from moiré and stack structures, which we take as an indication that more than one layer must be rotated in order to reach the limiting value of the mean free path achieved in the stack structures.

We demonstrate that an entropy measure based on a simple order parameter for the stacking (dis)order yields qualitative agreement with the angular dependence of the through-plane LTC in the moiré structures. This type of disorder is related to the moiré reconstruction, which in turn can be related to the shape of the slip surface and the layer stiffness. This strongly suggests that these quantities can be used as indicators for the efficacy of interlayer rotations as a means of reducing through-plane LTC.

Overall, the present results show that interlayer rotations can provide a chemistry-agnostic approach to controlling the through-plane LTC and the anisotropy ratio.

## Methods

### Stacking Sequences

Let us briefly recapitulate the different stacking sequences supported by the three materials of interest in this study. In the case of BN and MoS_2_ one can distinguish five different bulk stackings; AA, AB, AA′, AB1′, and AB2′, as exemplified in Figure 5a,f for BN.^46^ In the case of carbon, only the AA and AB stacking sequences are symmetrically unique, where the latter is also known as Bernal stacking.

In the AA and AA′ stackings, all atoms have neighbors directly above and below. These stackings can also be classified as open (alternatively sparse or eclipsed), and the hexagonal structure is clearly apparent. In the AA stacking, B is on top of B and N is positioned on top of N. In the AA′ stacking, on the other hand, different atom types are stacked on top of each other.

The AB, AB1′, and AB2′ stackings can be classified as closed (alternatively dense or staggered). Due to the lack of an inversion center for BN and MoS_2_ there are three variants. The AB stacking can be thought of as B on top of N with N and B placed in alternating hexagons. The primed AB structures, AB1′ and AB2′, feature the same atom type on top of each other, whereas the respective other type resides inside the hexagons.

The primed and unprimed structures cannot be related to each other via a simple translation of one layer but are instead related by a 60° rotation of one layer in combination with a translation. This results in two different types of slip surfaces. For carbon, the AB stacking is energetically the most favorable. For BN and MoS_2_ the lowest energy structures AA′ and AB are very close in energy^46^ where the energetic ordering is sensitive to the level of theory, including the choice of XC functional. Therefore, for BN and MoS_2_ we consider both AA′ and AB stackings throughout the paper. While there is only one ground structure for each material, the other stacking sequences appear in the reconstructed moiré structures due to geometric constraints.

### Construction of MLPs

We employed the second (NEP2)-^47^ and third-generation (NEP3) NEP scheme^48^ to build MLPs for C (NEP2), BN (NEP3), and MoS_2_ (NEP3) using the GPUMD package.^48−50^ The CALORINE^51^ and ASE^52^ packages were used to construct the NEPs, handle atomic structures, and set up MD simulations.

The NEP model uses a multilayer perceptron neural network architecture with a single hidden layer. The radial part of the atomic environment descriptor is constructed from linear combinations of Chebyshev basis functions, while the three-body angular part is similarly built from Legendre polynomials. For the radial part cutoffs of 8, 8, and 7 Å are used for C, BN, and MoS_2_, respectively. For the angular part cutoffs of 3.5, 4, and 4 Å are used for C, BN, and MoS_2_, respectively. The hidden layer contains 50 neurons for all systems.

The NEP models were trained by using a bootstrapping procedure in combination with active learning. The initial training set included primitive structures of the different stackings both strained and unstrained as well as moiré structures up to moiré index 6 (corresponding to an angle of about 5°) both fully relaxed and with random displacements generated using the Monte Carlo rattling procedure implemented in the HIPHIVE package.^53^ Further structures were generated by MD simulations run at temperatures between 100 and 900 K of both bulk and moiré structures and added to the training set over a few iterations (Table S1). For model validation see Figures S1–S5.

Lastly, we also employed the BOP model for MoS_2_^40,41^ used in ref (10) for comparison with our NEP model.

### DFT Calculations

The energy, forces, and virials for the training structures were obtained via DFT calculations that were carried out using the projector augmented-wave method^54^ as implemented in the Vienna ab initio simulation package.^55,56^ The XC contribution was represented using the vdW-DF-cx method.^57,58^ For C we also carried out calculations using the PBE+D3(BJ)^59−61^ and the SCAN functionals.^62^ The Brillouin zone was sampled using a Γ-centered grid with a linear k⃗-point spacing of about 0.25 Å^–1^ and Gaussian smearing with a width of 0.1 eV. For the calculation of the forces, a finer support grid was employed to improve their numerical accuracy. All calculations were carried out using a plane-wave energy cutoff of 520 eV.

### Thermal Conductivity via GK

The GK method was used to calculate the LTC as implemented in the GPUMD package. Specifically, the equilibrium molecular dynamics (EMD) method was employed, and for each structure and temperature, 100 independent production runs with a length of 1 ns were performed in the microcanonical (NVE) ensemble. The simulations were equilibrated for 100 ps in the canonical (NVT) ensemble using the Langevin thermostat.^63^ The heat current was sampled every 10 fs, and the running thermal conductivity was extracted at 500 ps using the Helfand–Einstein method.^64−66^ The equilibrium lattice parameters for each temperature were found via isobaric–isothermal (NPT) MD simulations using stochastic velocity^67^ and cell rescaling.^68^ A time step of 1 fs was used for all simulations.

The simulations of the stack systems were performed using a 2 × 2 × 2 supercell for a total of 63984 atoms in the case of C and BN and a total of 95976 in the case of MoS_2_. For the moiré simulations the repetition was *N* × *N* × 6 (i.e., 12 monolayers), where *N* varied depending on the index of the moiré cell so that the total number of atoms stayed above approximately 23000.

### Mode Projection

We employed phonon mode projection in order to analyze the phonons in the bulk and the stack systems from MD simulations.^69−71^ The modes analyzed include the LA, LO, TA, and TO modes along Γ → A. For the stack system, the bulk phonon modes were used, and although these are not exact harmonic eigenmodes of the system, they are good approximations. The autocorrelation function of the mode projected coordinate and velocity were fitted to damped harmonic oscillator functions in order to extract the frequencies and lifetimes of the LA and LO modes.^72,73^ MD simulations were run in the microcanonical ensemble (NVE) for 1 ns, and results were averaged over about 50 independent runs. These simulations were run using 40 monolayers for all systems.

### LTC from PBTE

The phonon dispersions of the ideal bulk structures were also calculated using the PHONOPY package.^74^ The LTC for the ideal bulk structures was calculated using the direct solution of a linearized phonon Boltzmann equation as implemented in the PHONO3PY package.^75^ The force constants were obtained in a 6 × 6 × 3 supercell, and the LTC was calculated using a 30 × 30 × 10 *q*-point mesh.

### Stacking Order Parameter

To measure the stacking (or out-of-plane) disorder in moiré structures, we introduce a simple atomic order parameter α_*i*_, which for atom *i* is defined as
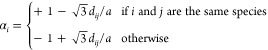
3where
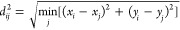
is the shortest in-plane distance between atom *i* and any atom *j* in the neighboring layer. For the bulk stacking sequences (Figure 5a,f) one obtains α = −1, 0, 1, whereas for the moiré structures α adopts continuous values between −1 and +1. The stacking (or out-of-plane) disorder can be estimated via the entropy as defined in eq 2.

### Moiré Structures

The moiré structures were constructed according to the method described in ref (76). For all three materials, moiré indices 1–11, 14, 22, 32, 45, 60, and 85 were included, corresponding to twist angles ranging from 21.8 to 0.39°. For BN and MoS_2_ two sets of moiré structures were constructed corresponding to the two distinct slip surfaces (slip surface).

### Stack Structures

The rotational disordered stacks were constructed by restricting the allowed in-plane strain of each layer to less than 1%. Each of the 10 layers contains approximately 400 primitive monolayer cells corresponding, e.g., to a 20 × 20 × 5 AB stacked graphite supercell.^77^ The twist angles between the layers are 0, 1.44, 4.31, 7.15, 12.52, 17.48, 22.85, 25.05, 25.69, and 28.56°. The stacks used here are slightly different from the ones used for simulations in ref (10), but the results (Figure 2c) show this to have an indiscernible effect on the thermal conductivity.

## Data Availability

The NEP models and databases of the DFT calculations as well as associated scripts are available on Zenodo (10.5281/zenodo.7811020).

## Supporting Information Available

Simplified model for collapsed transverse modes and their effect on the LTC, parity plots for energy, forces, and virials for NEP models, binding energy curves, phonon lifetimes for LA and LO modes, lattice thermal conductivity using different XC functionals, demonstration of effect of repetition length on through-plane lattice thermal conductivity in C, and overview of data used for training NEP models (PDF)
