# Supplementary material for: Tuning the Through-Plane Lattice Thermal Conductivity in van der Waals Structures through Rotational (Dis)ordering
Source: ACS Nano. 2023 Dec 8;17(24):25565–74. doi: 10.1021/acsnano.3c09717 (PMC10753894; doi:10.1021/acsnano.3c09717)
Supplement: Supplementary file 1 — nn3c09717_si_001.pdf [file nn3c09717_si_001.pdf]

Supporting Information

Tuning the through-plane lattice thermal conductivity in van-der-Waals structures through rotational (dis)ordering

Fredrik Eriksson[1], Erik Fransson[1], Christopher Linderälv[1], Zheyong Fan[2], and Paul Erhart[1,*]

[1] *Department of Physics, Chalmers University of Technology, SE-412 96 Gothenburg, Sweden*
[2] *College of Physical Science and Technology, Bohai University, Jinzhou 121013, P. R. China*
[*] *erhart@chalmers.se*

Contents

Supplementary Notes **2**
S1. Simplified modeling of collapsed transverse modes 2

Supplementary Figures **3**
S1. Parity plots for carbon NEP model based on the vdW-DF-cx functional 3
S2. Parity plots for the carbon NEP model based on the PBE+D3(BJ) functional 3
S3. Parity plots for the carbon NEP model based on the SCAN functional 3
S4. Parity plots for the BN NEP model based on the vdW-DF-cx functional 4
S5. Parity plots for the MoS_2 NEP model based on the vdW-DF-cx functional 4
S6. Binding energy curves for C 4
S7. Binding energy curves for BN and MoS_2 5
S8. Phonon lifetimes for LA and LO modes 6
S9. Lattice thermal conductivity using different XC functionals 7
S10. Effect of collapsing TA mode on phonon dispersion and lattice thermal conductivity . . 8
S11. Effect of repetition length on through-plane lattice thermal conductivity in C 9

Supplementary Tables **10**
S1. Overview of data used for training NEP models 10

Supplementary References **10**

Supplementary Notes

Supplementary Note S1: Simplified modeling of collapsed transverse modes

Let us set up a simple model system that allows us to show that the reduction in lifetimes of the longitudinal acoustic mode and the consequential drop in the through-plane lattice thermal conductivity (LTC), can be directly related to the collapse of the transverse acoustic (TA) modes.

To this end, we compute the through-plane LTC of graphite at 300 K in the framework of the Peierls-Boltzmann transport equation (PBTE) using our neuroevolution potential (NEP) model based on the van-der-Waals density functional with consistent exchange (vdW-DF-cx) (Table S1) to obtain the second and third-order force constants using HIPHIVE.[1] We introduce a scaling factor, λ, that allows us to systematically reduce the frequency of the TA mode and lowermost transverse optic (TO) modes along the $\Gamma - \mathrm{A}$ direction while all other parameters including the third-order force constants are left unchanged (Fig. S10).

This approach allows us to control which scattering processes are allowed by energy and momentum conservation laws without changing the efficiency of these processes as described by the third-order force constants. The results show that a reduction of the frequency of the TA modes from about 5 meV to 1 meV leads to a drop of the through-plane LTC by a factor of two, while a reduction to 0.3 meV gives rise to a reduction by a factor of ten Fig. S10. This effect can be understood by realizing that the collapse of the transverse modes substantially increases the space of allowed scattering processes as there are now many more modes of the same energy but different momenta. While this model is very simplified it nonetheless demonstrates the importance of the collapsing transverse modes on thermal transport even if the modes themselves are not major heat carriers.

We train the harmonic force constants from small displacements structures plus a set of structures for which only the TA/TO modes are active. The forces in these latter training structures can be artificially re-scaled with a factor λ (smaller than 1) when training, which effectively will lead to harmonic force constants with softer TA/TO modes while all other modes remain unchanged Fig. S10. These harmonic force constants are then used in PBTE calculations using PHONO3PY[2] with the same set of third-order force constants (obtained by PHONO3PY) for all calculations.

The obtained thermal conductivity clearly demonstrates a connection between the softening of the shear modes (TA, TO) and the decrease in through-plane thermal conductivity (Fig. S10). Lastly, we note that the shear modes in BN do not soften as much in the case of carbon, and BN does not exhibit as large of a reduction in thermal conductivity as carbon, in good agreement with this reasoning.

Supplementary Figures

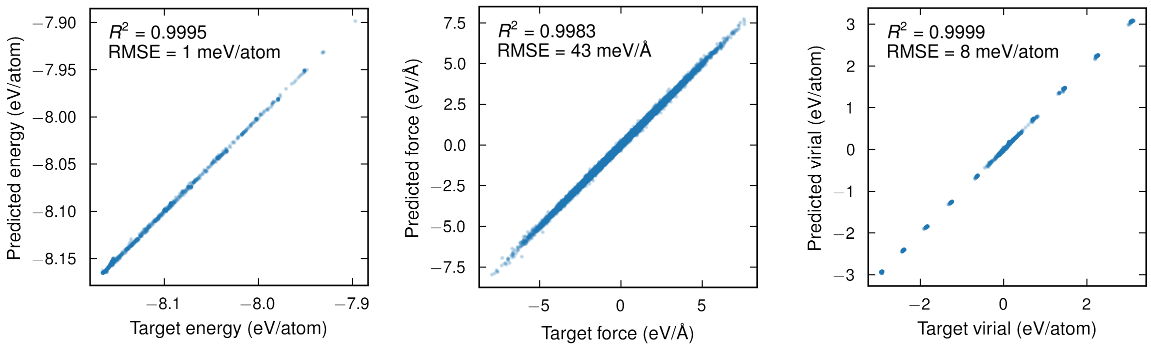

Figure S1: **Parity plots for carbon NEP model based on the vdW-DF-cx functional.**

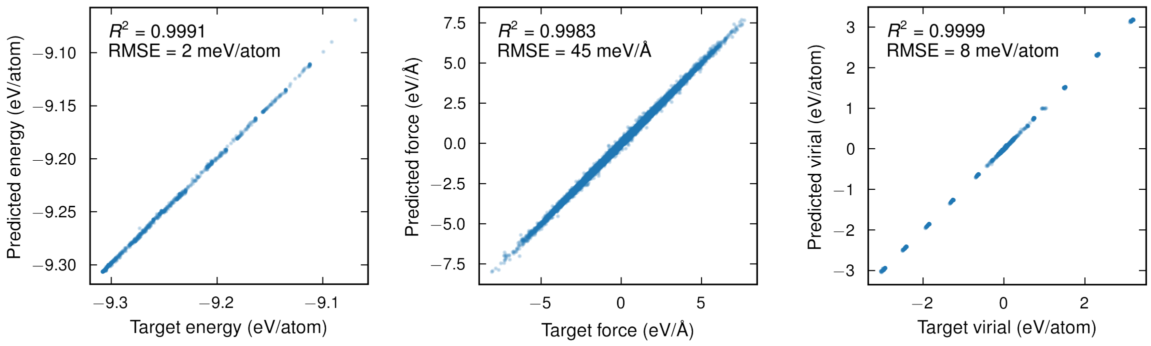

Figure S2: **Parity plots for the carbon NEP model based on the PBE+D3(BJ) functional.**

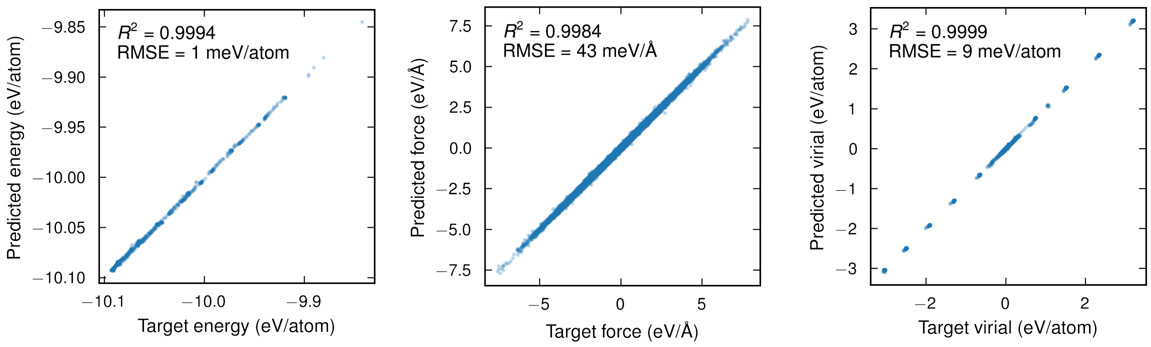

Figure S3: **Parity plots for the carbon NEP model based on the SCAN functional.**

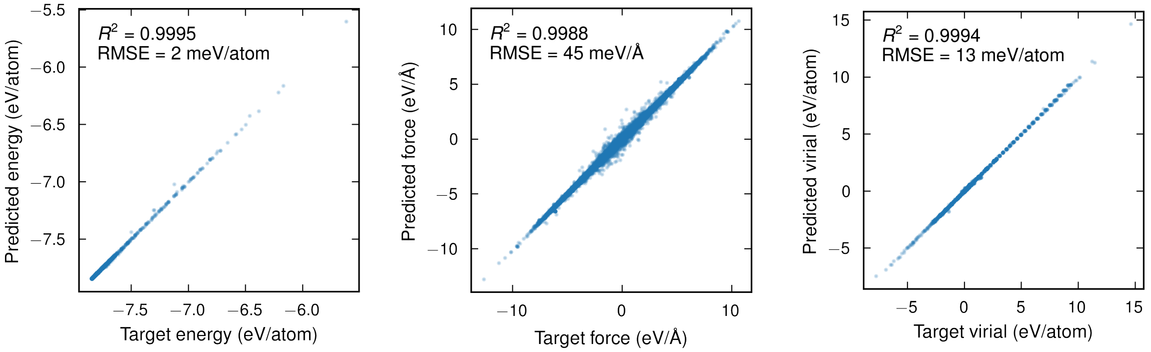

Figure S4: **Parity plots for the BN NEP model based on the vdW-DF-cx functional.**

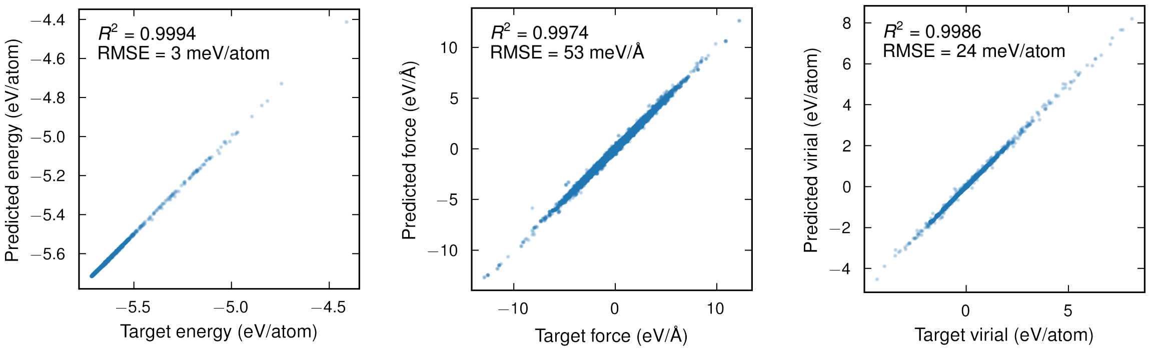

Figure S5: **Parity plots for the MoS_2 NEP model based on the vdW-DF-cx functional.**

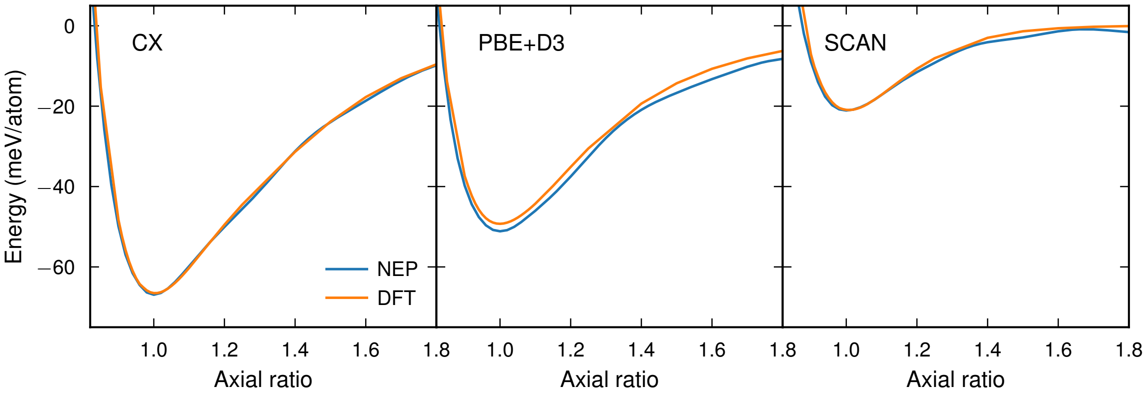

Figure S6: **Binding energy curves for C.**

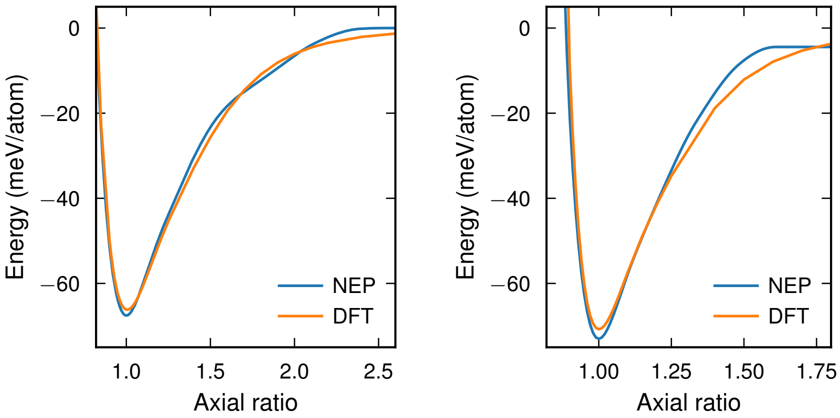

Figure S7: **Binding energy curves for BN and MoS_2.**

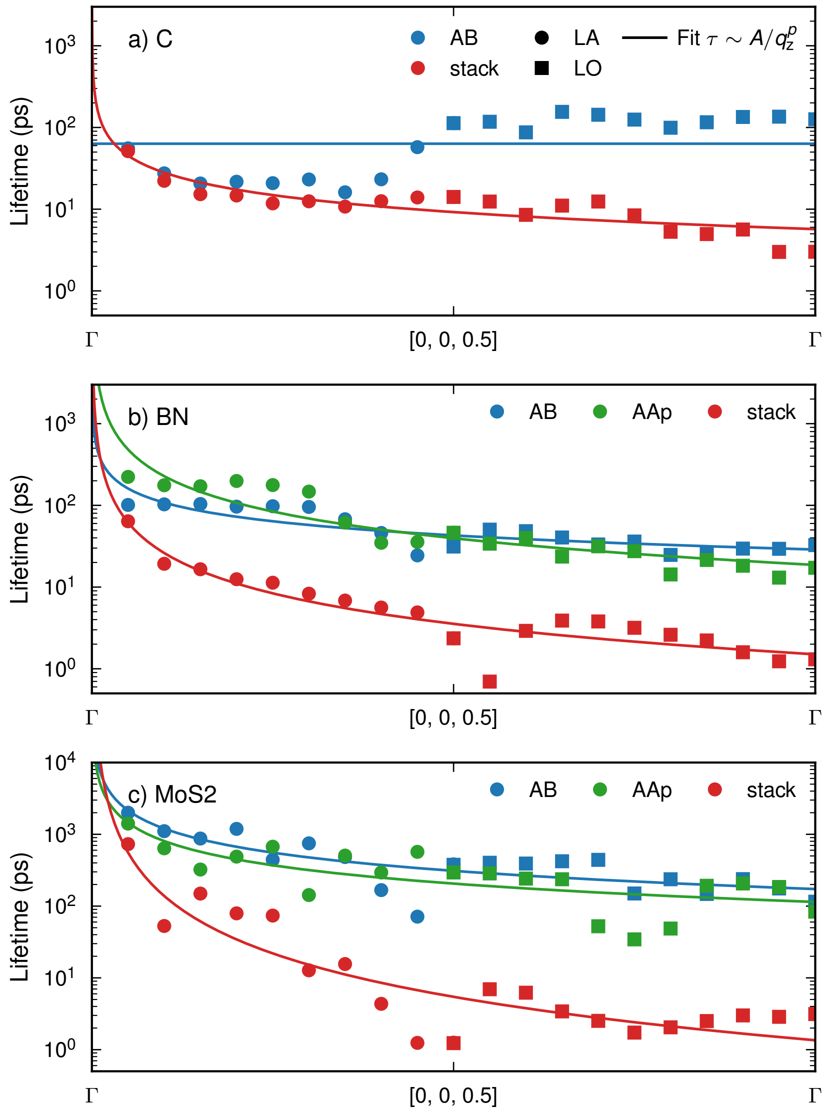

Figure S8: **Phonon lifetimes for LA and LO modes.** Data for (a) C, (b) BN, and (c) MoS_2 for ideal bulk and rotationally disordered stacks at 300 K. Here, the optical modes are shown as an unfolded continuation of the $\Gamma \rightarrow$A path.

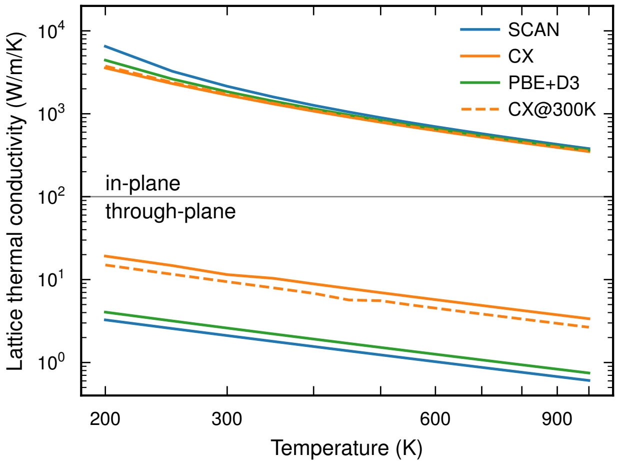

Figure S9: **Lattice thermal conductivity using different XC functionals.** Calculations were carried out for C within the framework of the PBTE. The dashed orange line illustrates the effect of thermal expansion showing the results at the 300 K lattice parameters from the NEP model constructed using the vdW-DF-cx functional. This highlights the effect of the lattice parameters and the sensitivity to the choice of XC functional. The 0 K lattice constants for PBE+D3, vdW-DF-cx, and SCAN are 6.97, 6.56, and 6.93 Å. The 300 K lattice constant were 6.60 Å. For reference the rotationally disordered stack has an effective lattice constant of 6.74 Å at 300 K. The calculations were performed using a $30 \times 30 \times 10$ $\boldsymbol{q}$-point mesh with force constants from a $6 \times 6 \times 3$ supercell.

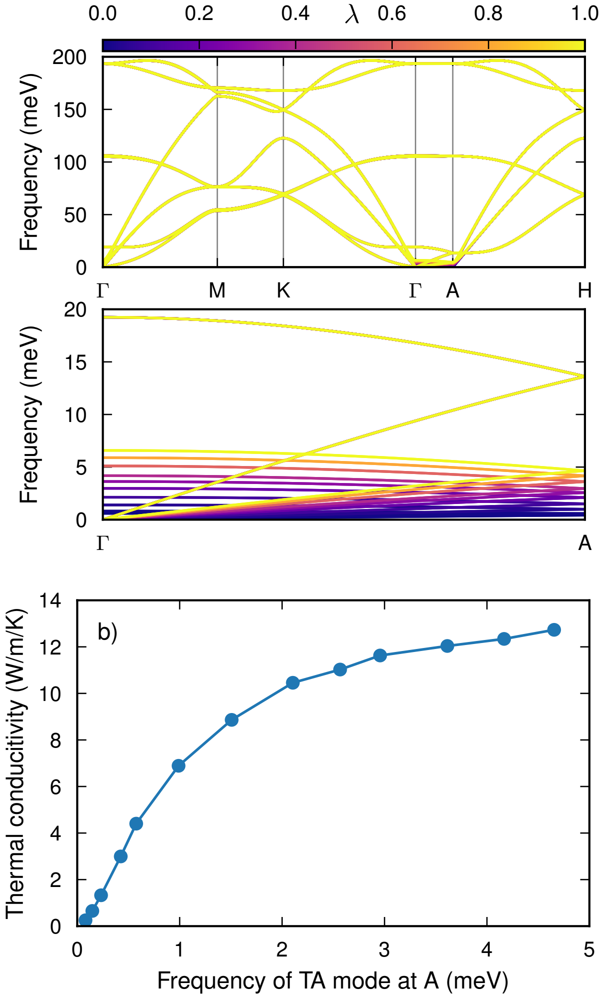

Figure S10: **Effect of collapsing TA mode on phonon dispersion and lattice thermal conductivity.** Left: Phonon dispersions for graphite with rescaled TA branch, where $\lambda = 1$ corresponds to the unchanged second-order force constants. Right: Through-plane LTC of graphite from PBTE at 300 K obtained using the phonon dispersions shown on the left combined with the third-order force constants of the unmodified system ($\lambda = 1$).

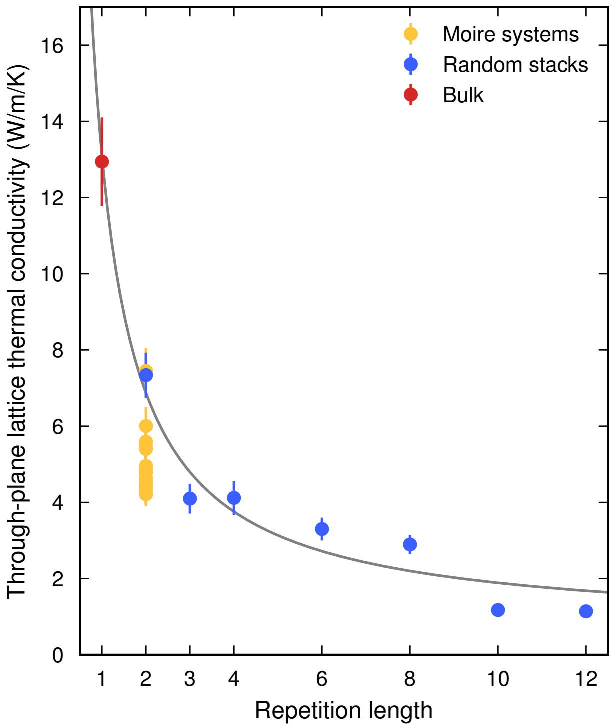

Figure S11: **Effect of repetition length on through-plane lattice thermal conductivity in C.** Each structure was created by drawing random layers from the 10-layer prototype stack with replacement. Here, the ideal graphite AB bulk structure is shown as repetition "1" and a repetition sequence of 2 corresponds to the bulk moiré structures. For low repetition lengths the variance is large as indicated by the moiré structures. The 10-layer data point is the stack structure used with all 10 unique layers. A 24-layer stack gave identical results to the 10 and 12-layer stacks. The error bars are the statistical uncertainty from the Green-Kubo method which is roughly proportional to the conductivity independent of stack configuration.

Supplementary Tables

Table S1: **Overview of data used for training NEP models.**

Material	XC	Model type	Number of structures	Total number of atoms
C	vdW-DF-cx	NEP2	1159	122 052
	PBE+D3(BJ)	NEP2	1133	116 990
	SCAN	NEP2	1089	108 474
BN	vdW-DF-cx	NEP3	8797	206 026
MoS_2	vdW-DF-cx	NEP3	2002	129 855

Supplementary References

[1] Fredrik Eriksson, Erik Fransson, and Paul Erhart. The Hiphive Package for the Extraction of High-Order Force Constants by Machine Learning. *Advanced Theory and Simulations*, 2:1800184, 2019.

[2] Atsushi Togo, Laurent Chaput, and Isao Tanaka. Distributions of phonon lifetimes in brillouin zones. *Physical Review B*, 91:094306, Mar 2015. doi: 10.1103/PhysRevB.91.094306.
